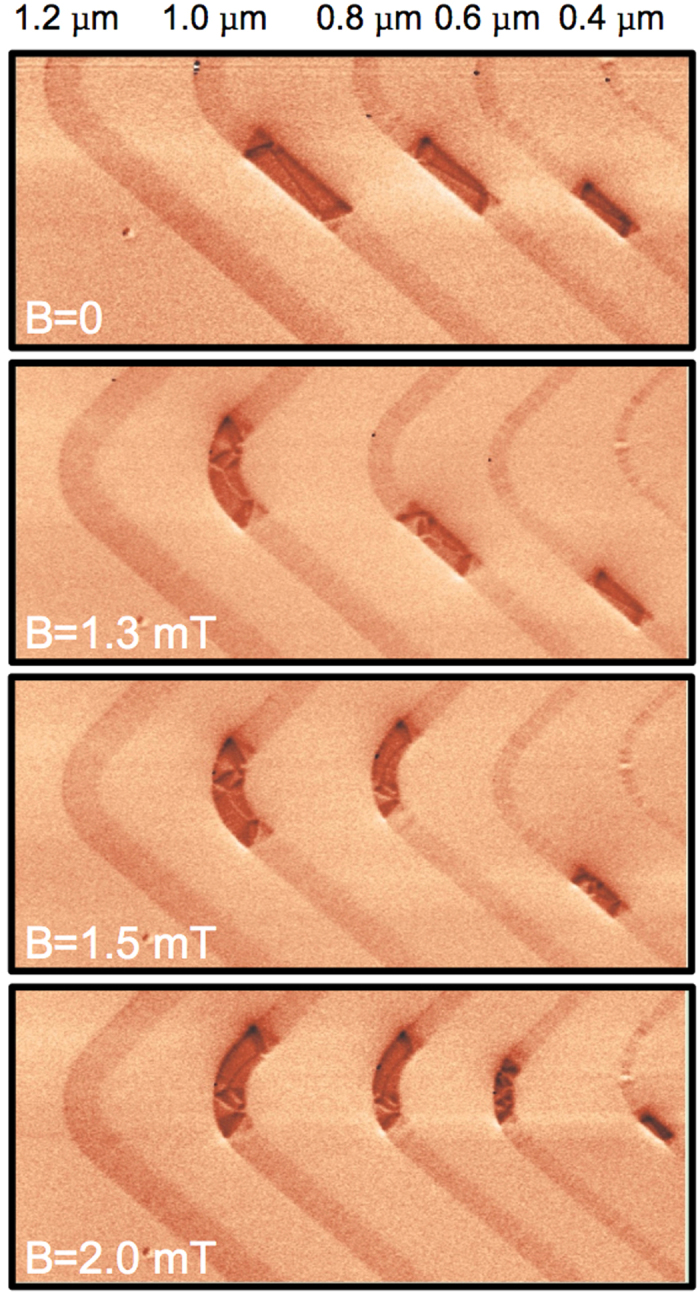# Third type of domain wall in soft magnetic nanostrips

**DOI:** 10.1038/srep12417

**Published:** 2015-07-23

**Authors:** V. D. Nguyen, O. Fruchart, S. Pizzini, J. Vogel, J.-C. Toussaint, N. Rougemaille

**Affiliations:** 1 CNRS, Inst NEEL, F-38000 Grenoble, France; 2 Univ. Grenoble Alpes, Inst NEEL, F-38000 Grenoble, France

## Abstract

Magnetic domain walls (DWs) in nanostructures are low-dimensional objects that separate regions with uniform magnetisation. Since they can have different shapes and widths, DWs are an exciting playground for fundamental research, and became in the past years the subject of intense works, mainly focused on controlling, manipulating, and moving their internal magnetic configuration. In nanostrips with in-plane magnetisation, two DWs have been identified: in thin and narrow strips, transverse walls are energetically favored, while in thicker and wider strips vortex walls have lower energy. The associated phase diagram is now well established and often used to predict the low-energy magnetic configuration in a given magnetic nanostructure. However, besides the transverse and vortex walls, we find numerically that another type of wall exists in permalloy nanostrips. This third type of DW is characterised by a three-dimensional, flux closure micromagnetic structure with an unusual length and three internal degrees of freedom. Magnetic imaging on lithographically-patterned permalloy nanostrips confirms these predictions and shows that these DWs can be moved with an external magnetic field of about 1 mT. An extended phase diagram describing the regions of stability of all known types of DWs in permalloy nanostrips is provided.

Magnetic domain walls in nanostructures present a range of properties allowing to consider them as objects in their own right. They carry a magnetic charge, have a chirality or a circulation^1^,^2^,^3^ and can show inertia^4^,^5^,^6^. The domain wall internal structure depends on the thickness, width and material of the constituting magnetic nanostructure^1^,^7^,^8^. This internal structure has a strong influence on the DW dynamics, which is of uttermost importance for devices in which the domain wall position is controlled and manipulated by field^9^,^10^,^11^,^12^,^13^ or current pulses^14^,^15^,^16^.

Micromagnetic simulations have proven to be a powerful tool to predict domain wall configurations in confined systems, as well as their dynamic behavior^1^,^7^,^17^. For flat strips of soft magnetic materials with in-plane magnetisation, like permalloy (Py), simulations showed the existence of two types of domain walls, the so-called transverse (TW) and vortex (VW) domain walls. A phase diagram was determined, predicting the regions of stability of both walls in a typical thickness range of 0–50 nm and widths 100–600 nm^1^,^7^. This phase diagram was recently extended to much wider strips and double/triple vortex configurations were found to be low-energy states^18^.

Using micromagnetic simulations and Magnetic Force Microscopy (MFM) measurements, we investigate the magnetic properties of domain walls in the case of Py strips thicker than 50 nm, for which the vortex wall is expected to be energetically favored on the basis of the existing phase diagram^1^,^7^,^17^. However, above a critical thickness *t*_C_ ≈ 60 nm, and almost independently of the width of the strip, we find that the vortex wall transforms into a flux closure configuration stretched along the length of the strip, with in-plane magnetisation curling around a Bloch wall of finite length. This prediction is confirmed experimentally by MFM images obtained on a series of 80 nm-thick Py strips of various widths. The observed domain walls show two unusual features: i) contrary to usual TWs and VWs, they are significantly longer than the width of the strip, ii) they are characterised by three internal degrees of freedom.

## Results

**Figure 1:**
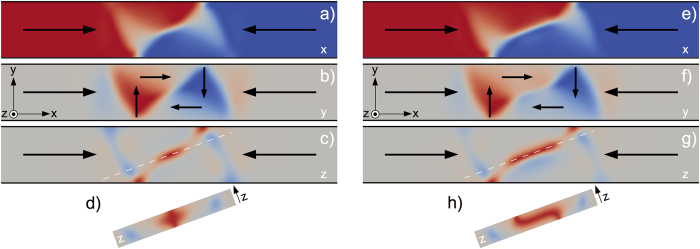
Micromagnetic configuration of a head-to-head domain wall in a 200 nm wide, 60 nm thick Py strip. (**a**–**d**) Vortex domain wall. (**e**–**h**) Landau domain wall. In all images, X, Y and Z stand for the length, width and thickness of the strip, respectively. The red/blue color code gives the amplitude of the magnetisation component along X (**a**,**e**), Y (**b**,**f**) and Z (**c**,**d**,**g**,**h**). Black arrows represent the local direction of magnetisation. (**a**–**c**,**e**–**g**) XY cross section views of the domain walls taken at mid-height (z = 30 nm). (**d**,**h**) Cross section views of the same domain walls along the dashed white line shown in (**c**) and (**g**).

We first consider, numerically, a 200 nm wide, 60 nm-thick Py strip. Two configurations can be obtained, as shown in Fig. 1: a vortex wall [Fig. 1(a–d)] and another flux closure magnetic state [Fig. 1(e–h)]. Contrary to the vortex wall in which the magnetisation distribution can be essentially considered as uniform within the thickness of the strip, the magnetisation distribution in this second DW varies significantly across the thickness. Figure 1(d,h) are cross section views illustrating this distribution: the micromagnetic configuration reveals that the DW is essentially a vortex with a core stretched along the length of the strip, i.e. a flux closure magnetic configuration curling around a Bloch wall instead of a vortex core. The magnetic flux is entering and exiting this Bloch wall at different locations of the domain wall, introducing an asymmetry compared to what is observed in a vortex core [see Fig. 1(d,h)]. The overall micromagnetic configuration is similar to a rectangular Landau flux-closure pattern^19^,^20^,^21^. In the following, we therefore refer to the Landau domain wall when describing this type of DW.

Investigating the influence of the width *w* of the strip on the critical thickness *t*_C_ at which the VW/Landau DW transition occurs, we find that *t*_C_ is essentially independent of *w* and of the order of 55 nm. This observation, together with the value found for *t*_C_, suggests that this transition may be linked to the Néel - Bloch wall transition observed in continuous films, which is known to occur at a film thickness of a few tens of nanometers, around 40 nm in Py^22^,^23^,^24^. Besides, the tilted feature of the Bloch part of the Landau wall is consistent with the known zigzag structure of head-to-head domain walls in thin films with in-plane uniaxial anisotropy^25^,^26^. In both systems, the tilt allows spreading the magnetostatic charges over a larger area, and thus decreasing the magnetostatic energy. In strips, the anisotropy is of dipolar origin (shape anisotropy), while it is of magnetocrystalline origin in films.

**Figure 2:**
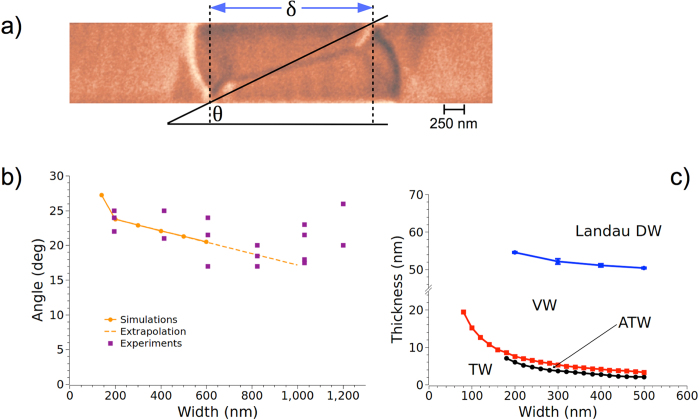
(**a**) MFM image of a head-to-head domain wall in a 1 μm wide, 80 nm thick Py strip. (**b**) Predicted angle *θ* for different width of a 80 nm thick Py strip (yellow dots). The dashed line is an extrapolation of the *θ* decrease as the width of the strip becomes larger. The purple squares are experimental data points deduced from the MFM images of 80 nm thick Py strips. (**c**) Extended phase diagram of Py strips showing the regions where the TW, asymmetric TW (ATW), VW and Landau DW have the lowest energy. The red and black data points are reproduced from Ref. 7.

To check these predictions, we fabricated a series of 40 and 80 nm-thick Py strips with different widths, ranging from 200 to 1200 nm. The strips are made with a curved shape to easily set a domain wall in the bent region after the application of an external magnetic field, transverse to the strip^27^,^28^. The resulting magnetic configuration is then imaged after moving the DW in the straight region of the strip. Consistent with micromagnetic simulations, we always observe Landau DWs in the thickest (80 nm) strips and conventional VWs in the thinnest ones (40 nm). An example of a MFM image for a 1 μm-wide Py strip is reported in Fig. 2(a). Note, in that image, the black magnetic contrast atop the Bloch wall crossing the entire DW and the unusual length of the overall micromagnetic configuration.

## Discussion

When increasing the thickness of the nanostrip, we observe numerically and experimentally an increase of the domain wall width. This result is expected as the amount of volume magnetic charges within the DW increases with the thickness of the strip. Using the MFM images, we measure the angle *θ* made by the long axis of the strip and the line passing through the points where the magnetic flux, transverse to the strip, enters and exits the Landau DW [see Fig. 2(a)]. Comparison between the predicted DW angle *θ* and the experimental findings, is reported in Fig. 2(b). Although our experimental measurements are scattered, a fairly good quantitative agreement is found between the angle values deduced from the MFM images and the predictions from micromagnetic simulations. The scattering of the experimental data points is likely due to imperfections in the strips, such as edge/interface roughness or pinning sites that could slightly deform the DWs, or can be linked to the way the DWs have been prepared and moved prior to their imaging. We also observe discrepancies for the widest strips (for 1000 and 1200 nm-wide strips). We emphasize though that micromagnetic simulations are hardly achievable for such large widths and thicknesses, and understanding if this non-monotonic behavior is an experimental artefact or if it has a physical origin remains an open question. However, we want to stress that, above a certain critical thickness (about 50 nm experimentally), we always observe Landau domain walls, independently of the width of the strip (at least in the 200–1200 nm range probed in this work). There is no exception and experiments have been reproduced several times on different samples (see Figs 1 and 2 in supplementary materials). Micromagnetic simulations performed for Py strips with widths and thicknesses in the range of 200–600 nm and 40–70 nm, respectively, allow us to provide an extended phase diagram, describing the regions of stability of the TW, VW and Landau DW [Fig. 2(c)].

Interestingly, when the Landau DW is found numerically, it always has a lower energy than the VW. In other words, within the set of parameters we use in the micromagnetic simulations, we never find the Landau DW as a metastable state. For example, a VW is always found if the thickness *t* of the strip is set to 50 nm or less, even if the initial condition in the micromagnetic simulation is a Landau DW. These results indicate that Landau DWs may not persist as a metastable state and suggest a second-order transition, consistent with the existence of a breaking of symmetry. We emphasize though that characterizing the order of the VW/Landau DW phase transition deserves more investigations, beyond the scope of the present work.

**Figure 3:**
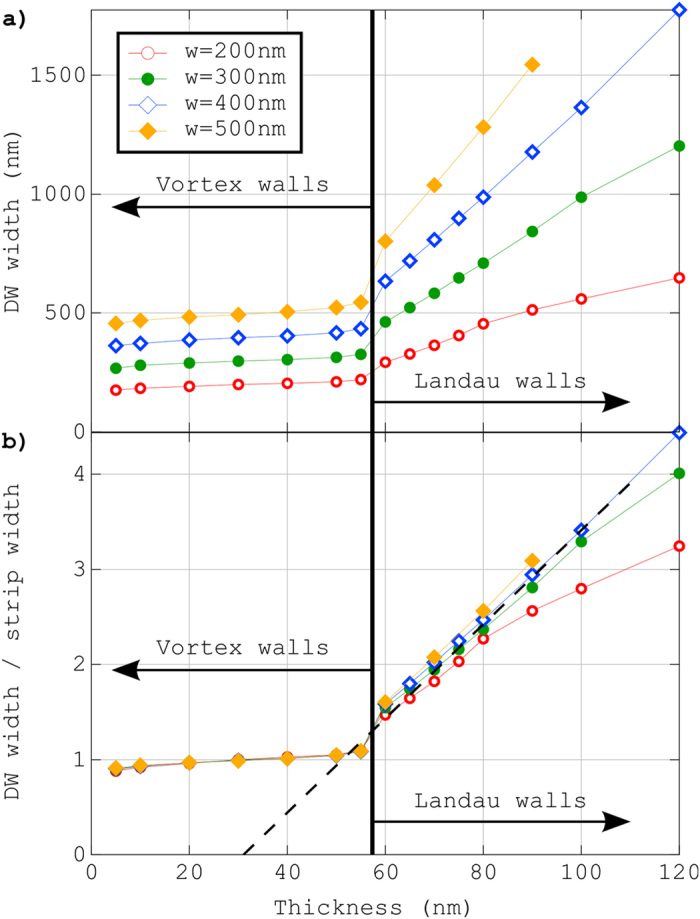
Domain wall width *δ* (**a**) and DW width divided by the strip width *δ*/*w* (**b**) deduced from micromagnetic simulations as a function of the strip thickness *t*. In (**b**), the black dotted line is the phenomenological scaling law: δ ≈ *w*(*t*–6Δ_d_)/(4Δ_d_).

**Figure 4:**
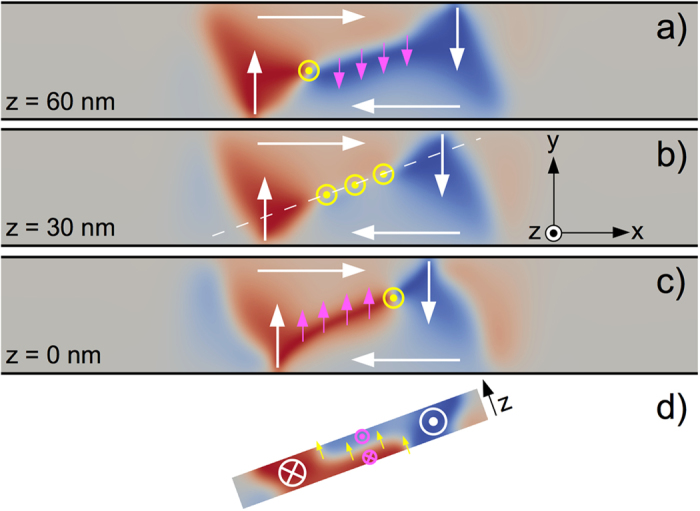
(**a**–**c**) XY cross section views of a Landau DW at different heights [(**a**) z = 60 nm, (**b**) z = 30 nm, (**c**) z = 0 nm] illustrating the 3 internal degrees of freedom the (clockwise) chirality (white arrows), the polarity of the Bloch wall (yellow arrows) and the direction of the Néel caps (magenta arrows) for the bottom (*z* = 0 nm) and top (*z* = 60 nm) surfaces. (**d**) Cross section view along the dashed white line shown in (**b**). In all images, X, Y and Z stand for the length, width and thickness of the strip, respectively. The red/blue color code gives the amplitude of the magnetisation component along Y.

Inspection of the dependence of the DW width *δ* as a function of both the strip width *w* and strip thickness *t* is also useful to compare quantitatively experiments and micromagnetic predictions. In the following, we define the DW width *δ *= *w*/tan *θ* [Fig. 2(a)]. Figure 3(a,b) show *δ* and *δ*/*w* as a function of the strip thickness *t*. The latter clearly reveals two phenomenological scaling laws: *δ* ≈ *w* for *t* < *t*_C_ and *δ* ≈ *w*(*t*–6Δ_d_)/(4Δ_d_) for *t* > *t*_C_, with 
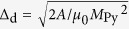
 the dipolar exchange length. The first scaling law simply reflects the double triangular shape of the VW, rather rigidly kept due to the internal 90° walls. The second one may be explained with a crude model accounting for the competition between dipolar and exchange energies, and assuming that the Bloch wall extends along the diagonal of the rectangular shape of the Landau DW. This model predicts *δ*~*wt* (see Method section), thus describing accurately the slope of the scaling law deduced from the simulations. The absence in the model of the horizontal shift 6Δ_d_ is thought to be linked to the length of the Bloch wall [see Fig. 2(a)], which is smaller than the full diagonal of the supposed rectangular shape of the Landau pattern. Also, the deviation at large *t* for narrow strips results from the ersatz used in the model to estimate the dipolar energy, assuming a thin strip. Qualitatively, the total length of the wall measured experimentally is well reproduced: while transverse and vortex walls have a typical size comparable to the width of the strip^1^,^7^, this Landau DW is significantly larger, two-three times the strip width, consistent with the simulations [Fig. 4(b)].

Landau DWs have additional interesting properties. Like vortex walls, they have two internal degrees of freedom: a chirality, associated with the clockwise or anticlockwise circulation of the in-plane magnetisation, and a polarity, associated with the out-of-plane direction of the magnetisation within the Bloch wall. But unlike the vortex walls, the Landau DW has a third internal degree of freedom associated with the in-plane direction of the top and bottom Néel caps (magenta arrows in Fig. 4). Similar to what is observed in thick, self-assembled Fe micro-dots^29^,^30^,^31^, the two Néel caps have their magnetisation pointing in opposite directions to minimize the magnetostatic energy of the asymmetric Bloch wall.

Finally, this entire, three-dimensional micromagnetic configuration can be moved under an applied magnetic field. This is illustrated in Fig. 5, where a field of 1 mT was applied along the strip length to move the DWs from the straight zone to the bent region of the strip, where they were first nucleated. Similar to what is often observed in thinner Py strips, all Landau DWs could be moved with a relatively small field, 1–2 mT typically. A closer inspection of the MFM images, before and after the application of the external magnetic field, reveals some changes in the internal micromagnetic configurations of the Landau DW. These changes are likely due to the domain wall dynamics during their motion. Understanding the magnetisation dynamics of Landau DWs deserves however more investigations.

## Conclusion

In summary, we have demonstrated experimentally and numerically the existence of a third type of magnetic domain wall in thick Py nanostrips. This wall is characterised by a three-dimensional micromagnetic structure with an in-plane flux closure configuration curling around a Bloch wall of finite length, similar to a Landau flux-closure pattern. The width of this Landau DW is unusually large and can significantly exceed a few microns in Py strips (see Figs 2 and 5). The Landau DW has also interesting properties: besides the chirality of the flux closure configuration and the polarity of the Bloch wall, the wall has well-defined directions for the Néel caps. The static and dynamic properties of this wall could open new prospects in nanomagnetism and field- or current-induced domain wall motion.

## Methods

Numerically, our approach is based on the finite difference OOMMF code^32^. Material parameters are *μ*_0_*M*_Py_ = 1.0053 T for the spontaneous magnetisation, *A*_Py_ = 10 pJ/m for the exchange stiffness, and zero magnetocrystalline anisotropy. This implies a dipolar exchange length 
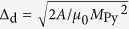
 ≈ 5 nm. Although directly applicable to Py, our simulations may be applied to any other magnetic material with no magnetocrystalline anisotropy, scaling lengths with Δ_d_. Cell size in all simulations presented here was set to 4 × 4 × 5 nm^3^. Using a cell size of 4 × 4 × 2.5 nm^3^ yields only negligible differences. Damping is set to 1 to speed up convergence, with no consequence on the result as we are interested in equilibrium states. Magnetic moments at the two extremities of the strips are fixed to avoid non-uniform magnetisation distributions at the edges, and the length of the strips is chosen such that the aspect ratio is at least larger than 10 to limit finite size effects.

Experimentally, the internal micromagnetic configuration of the domain walls gives rise essentially to a monopolar contrast. This is typical for Bloch walls or asymmetric Néel walls with a significant out-of-plane magnetisation within the core, while Néel walls would mainly lead to a dipolar contrast. The MFM contrast may be dark or bright in Fig. 2(a), consistent with the possibility to have either up or down perpendicular component of the magnetisation within the wall [see Fig. 1(a)].

To determine the width *δ* of the Landau domain walls, we use the following model to calculate the dipolar and exchange energies. Dipolar energy of a head-to-head DW (whatever its exact shape) is estimated from a 2D slab carrying the surface magnetic charge *σ* = 2*twM*_Py_/(*wδ*) = 2*tM*_Py_/*δ*. The resulting dipolar energy integrated in space is 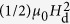, with *H*_d_ of the order of *σ*/2 at each surface of the strip and significant only above the wall over a distance *w*. The total dipolar energy is then of the order of 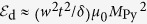. The Landau DW being bounded by two segments of wall, nearly perpendicular to the strip whatever the thickness and width (see Fig. 1), we neglect their contribution in the energy minimization. Most of the remaining energy is then associated with the tilted Bloch wall. The Bloch wall energy per unit length is mostly of exchange origin because of the rather good closure of the flux inside Bloch walls. For the thicknesses considered here, Bloch walls have a close-to-circular cross-section^33^,^34^ and thus have an area of the order of *t*^2^. This leads to an energy per unit length of *A*(*π*/*t*)^2^*t*^2^ = *Aπ*^2^. Finally, the Bloch wall is assumed to extend over the diagonal of the rectangular shape of the Landau wall, and its energy reads: 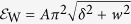. Minimization of the total energy versus *δ* provides the equilibrium value: 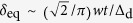. To obtain this value, we dropped the term related to *w*^2^ in the length of the Bloch wall, quickly being negligible above the transition thickness. As often for scaling laws, the numerical factor is not directly applicable, due to the crude approximations made in the model: 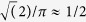, against 1/4 in the phenomenological law extracted from the simulations.

## Additional Information

**How to cite this article**: Nguyen, V. D. *et al.* Third type of domain wall in soft magnetic nanostrips. *Sci. Rep.* **5**, 12417; doi: 10.1038/srep12417 (2015).

## Supplementary Material

Supplementary Information

## Figures and Tables

**Figure 5:**