# Supplementary material for: Third type of domain wall in soft magnetic nanostrips
Source: Sci Rep. 2015 Jul 23;5:12417. doi: 10.1038/srep12417 (PMC5378877; doi:10.1038/srep12417)
Supplement: Supplementary Information [file srep12417-s1.pdf]

Supplementary materials.

Third type of domain wall in soft magnetic nanostrips

V. D. Nguyen,[1,2] O. Fruchart,[1,2] S. Pizzini,[1,2]
J. Vogel,[1,2] J.-C. Toussaint,[1,2] and N. Rougemaille[1,2,*]

[1] *CNRS - Institut NÉEL - F-38042 Grenoble - France*

[2] *Université Grenoble Alpes - Institut NÉEL - F-38042 Grenoble - France*

[*] *nicolas.rougemaille@neel.cnrs.fr*

(Dated: May 7, 2015)

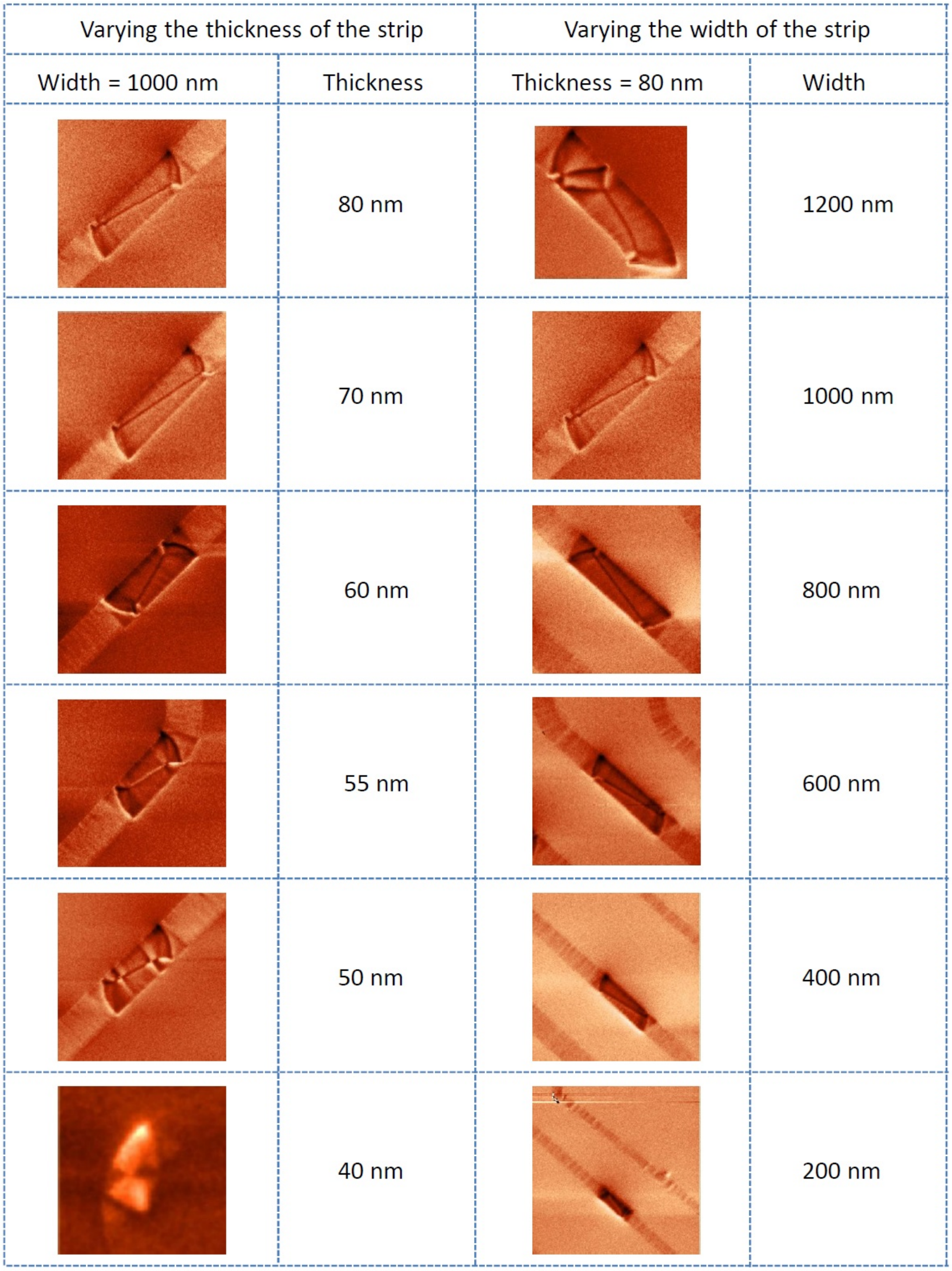

FIG. 1. (Color online) MFM images of Landau DWs in 1 µm-wide strips of different thicknesses (left column) and in 80 nm-thick strips of different widths (right column).

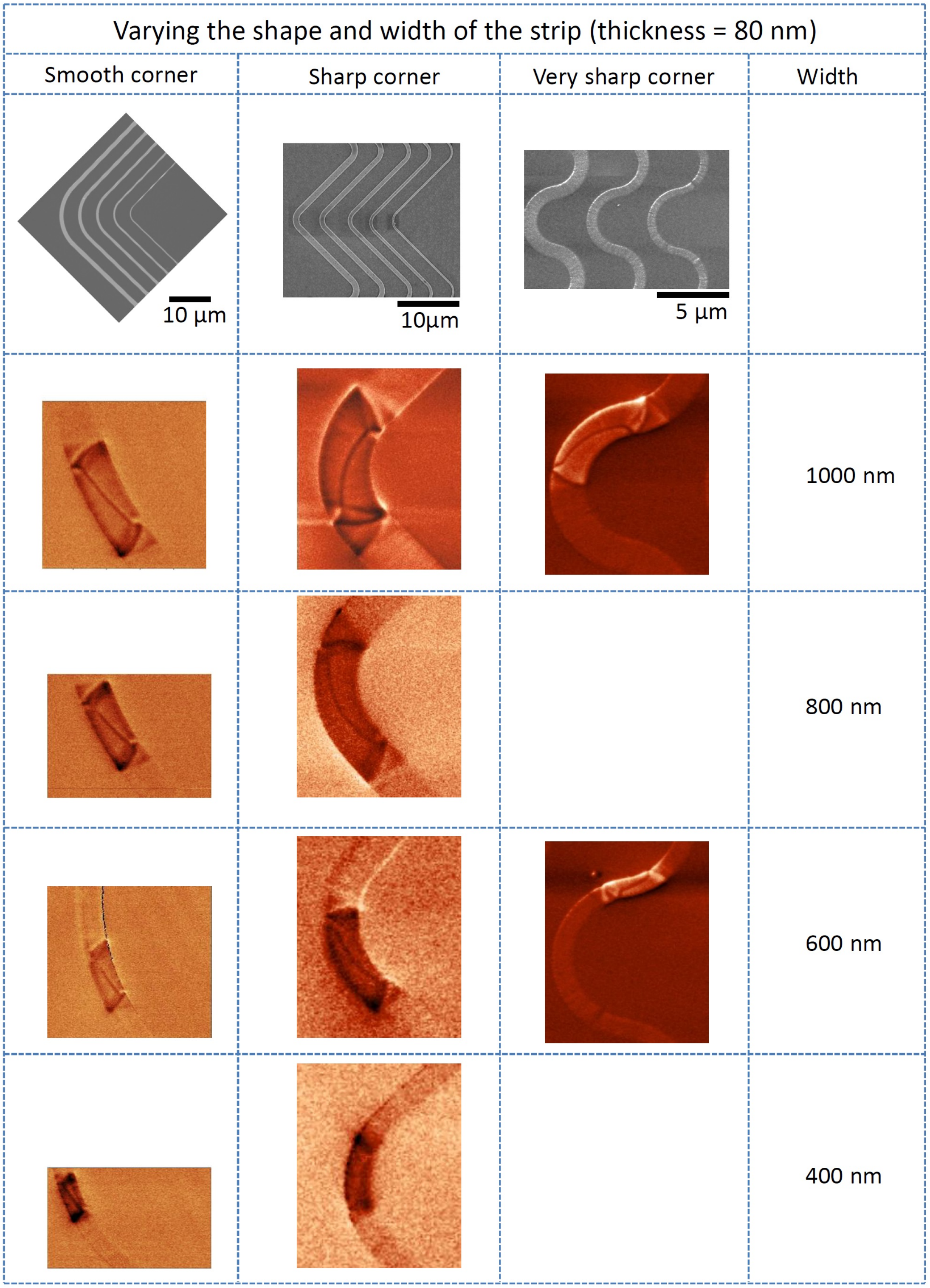

FIG. 2. (Color online) Electron micrographs and MFM images of Landau DWs in 80 nm-thick strips of different widths and shapes.
